# mTOR Regulation of Glycolytic Metabolism in T Cells

**DOI:** 10.3389/fcell.2018.00122

**Published:** 2018-09-25

**Authors:** Robert J. Salmond

**Affiliations:** Leeds Institute of Cancer and Pathology, St James’s University Hospital, University of Leeds, Leeds, United Kingdom

**Keywords:** T cell, aerobic glycolysis, mTOR, metabolism, immune responses

## Abstract

T cell activation, differentiation and effector function is intrinsically linked to the regulation of metabolic pathways. Evidence has shown that inflammatory T cell responses are dependent upon the adoption of aerobic glycolytic metabolism. Furthermore, activation and regulation of the mechanistic target of rapamycin signaling pathway serves a key determinant of T cell metabolism, with subsequent effects on T cell effector responses. In this mini-review, we discuss the mechanisms underpinning the function of the Warburg effect in T cell responses and the role of mTOR in these processes.

## Introduction

T cells serve as cellular effectors and orchestrators of adaptive immune responses during infection and cancer. In the past decade, a wealth of data has determined that T cell activation, clonal expansion, effector differentiation, and function is closely linked to and dependent upon the regulation of basic cellular metabolic processes. It has become clear that in effector T cells, the pyruvate produced by glycolysis is preferentially fermented to lactate even in the presence of oxygen; a classic example of the Warburg effect in non-transformed cells. In this review, we discuss how the engagement of aerobic glycolysis influences T cell activation and describe the role of the mechanistic target of rapamycin (mTOR) pathway in these processes.

## Metabolic Reprogramming During T Cell Activation

Prior to encountering antigen, T cells are quiescent and lack effector function. These naïve T cells uptake low levels of glucose and amino acids, and rely on mitochondrial oxidative phosphorylation (OXPHOS) to maintain cellular ATP levels [reviewed in Geltink et al. (2018)]. Naïve T cells may survive for years circulating through the blood and lymph, only rarely undergoing cell division. Upon encounter with peptide antigen-major histocompatibility complexes (MHC) presented by antigen-presenting cells, the differentiation of naïve CD4^+^ T cells to a plethora of specialized helper T cell (Th) subsets enables the immune system to respond appropriately to a huge variety of pathogens, from extracellular parasitic worms to intracellular viruses and bacteria. In this regard, CD4^+^ Th cells modulate the activity and function of innate and adaptive immune cells by secreting cytokines. CD4^+^ Th1 cells promote cell-mediated immunity by secreting interleukin (IL)-2, interferon (IFN)-γ and tumor necrosis factor (TNF) whereas Th2 cells promote humoral immunity through the production of IL-4, IL-5 and IL-13 (Asnagli and Murphy, 2001). Th17 cells produce high levels of IL-17 and are important for maintenance of homeostasis and protection from pathogens at barrier sites, such as the intestine (Stockinger and Omenetti, 2017). By contrast, regulatory CD4^+^ T cells (Treg), characterized by expression of the transcription factor forkhead box P3 (FOXP3), have a key role in limiting inflammation and preventing autoimmunity by suppressing the activity of other immune cell types (Sakaguchi et al., 2010). Cytotoxic CD8^+^ T cells have the capacity to target and kill infected and transformed cells, and produce inflammatory cytokines such as IFNγ (Halle et al., 2017). Upon resolution of an immune response, a number of memory T cell populations capable of responding rapidly to a second antigenic encounter are retained, facilitating life-long protection from re-infection.

The processes of T cell activation are bioenergetically expensive; for example, it has been estimated that, during infection, virus-specific CD8^+^ T cells undergo rapid proliferation with a population doubling time of only ∼8 h (De Boer et al., 2003). Therefore, a key question in immunology is: how do T cells fuel the processes of activation, proliferation and differentiation? Whereas cytokines such as IL-7 maintain low level glycolytic metabolism in naïve T cells (Jacobs et al., 2010), triggering of the T cell antigen receptor (TCR) by cognate peptide antigen-MHC presented on the surface of antigen-presenting cells, results in the upregulation of anabolic biosynthetic pathways in order to facilitate T cell activation. The integration of TCR, CD28 co-stimulation and cytokine receptor signals determines T cell metabolism and subsequently impacts upon differentiation, and effector function (Fox et al., 2005; Cornish et al., 2006; Jacobs et al., 2010; Michalek et al., 2011; Shi et al., 2011; Finlay et al., 2012; Gubser et al., 2013; Ray et al., 2015; Richer et al., 2015; Tan et al., 2017; Geltink et al., 2018). The regulation of aerobic glycolysis is central to these fate decisions.

## Aerobic Glycolysis Drives Effector T Cell Differentiation

An important role for glucose uptake, and glycolysis in T cell function was suggested four decades ago by the demonstration that the glycolysis inhibitor 2-deoxyglucose (2-DG) impaired T cell cytotoxic capacity (MacDonald, 1977; MacDonald and Cerottini, 1979). Furthermore, studies indicated that 2-DG treatment selectively reduced the expression of key effector molecules, including IFNγ and granzymes, and cell cycle proteins in both mouse (Cham and Gajewski, 2005; Cham et al., 2008) and human (Renner et al., 2015) CD8^+^ T cells. At low doses that do not impact upon TCR-induced proliferation, 2-DG also inhibits CD4^+^ Th2 (Yang et al., 2016) and Th17 (Shi et al., 2011) cell differentiation, but promotes Treg differentiation (Shi et al., 2011). Together, these studies indicate that the regulation of glycolytic flux plays a central role in cell fate decisions, and T cell differentiation. In recent years, mass-spectrometry based proteomic analyses have further informed our understanding of the extent to which the regulation of metabolic pathways is prioritized by T cells. Thus, studies from the Cantrell lab have shown that 41 glycolytic proteins represent 7% of the total protein molecules in effector cytotoxic CD8^+^ T cells (Hukelmann et al., 2016).

Upon TCR triggering, expression of plasma membrane glucose transporters is enhanced as part of the general process of metabolic reprogramming. T cell-specific knockout of the glucose transporter SLC2A1/GLUT1 substantially inhibited the activation of mouse CD4^+^ T cells (Macintyre et al., 2014). Whilst the homeostasis and survival of naïve T cells was unaffected by the absence of GLUT1, TCR-induced CD4^+^ T cell growth, and proliferation were profoundly impaired. Furthermore, differentiation of *Slc2a1^−/−^* T cells to effector Th1, Th2 and Th17, but not Treg, lineages was blocked (Macintyre et al., 2014), consistent with the known effects on T cell differentiation of inhibiting glycolytic flux with 2-DG. As anticipated, T cell activation defects in TCR-stimulated *Slc2a1*^−/−^ T cells were associated with reduced rates of glucose uptake, glycolysis and lactate production (Macintyre et al., 2014). The lack of a catastrophic impact of GLUT1-deficiency on glycolytic flux, and cell survival in naïve T cells is likely to be a consequence of expression of additional glucose transporters, including GLUT3, by T cells (Macintyre et al., 2014; Hukelmann et al., 2016). The importance of glucose uptake in T cell responses *in vivo* has been further highlighted by recent studies indicating that T cells and cancer cells directly compete for nutrients in the tumor microenvironment (Chang et al., 2015; Ho et al., 2015; Siska et al., 2017). Thus, highly glycolytic tumor variants suppress the activity of anti-tumor T cells, at least in part, by reducing the bioavailability of glucose.

**FIGURE 1:**
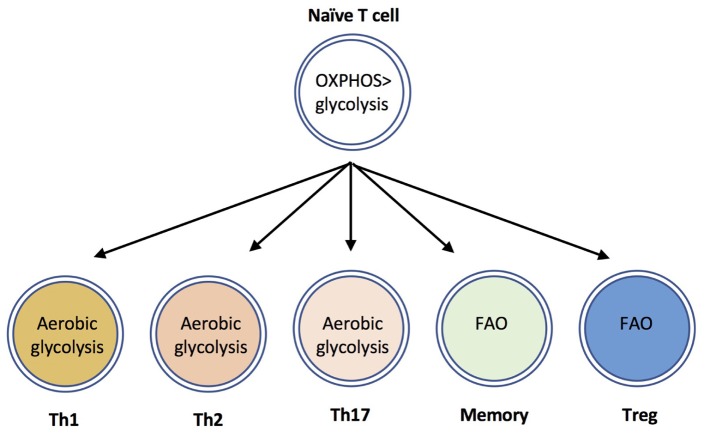
CD4^+^ T cell subsets use distinct metabolic programs. Naïve T cells uptake low levels of glucose and primarily utilize mitochondrial oxidative phosphorylation to maintain homeostasis. The differentiation and effector functions of inflammatory Th1, Th2, and Th17 cells relies on engagement of aerobic glycolysis. By contrast, memory T cells and Tregs are dependent upon fatty acid oxidation (FAO) pathways.

In addition to upregulating glycolytic metabolism, activated T cells also increase uptake and hydrolysis of amino acids such as glutamine, and modulate mitochondrial, and lipid metabolism [reviewed in Geltink et al. (2018)]. Distinct T cell populations differ in their utilization, and dependence upon these metabolic programs. Effector CD8^+^ T cells, and Th1, Th2 and Th17 CD4^+^ T cells are highly glycolytic, whereas Tregs are dependent upon fatty acid oxidation (FAO) (Michalek et al., 2011; Shi et al., 2011 Berod et al., 2014) (**Figure 1**). Based on the use of chemical inhibitors, FAO has also been suggested to be important for the development of memory T cells (reviewed in (Lochner et al., 2015)); although recent evidence using genetic mouse models suggest that the requirement for FAO is not absolute (Pan et al., 2017; Raud et al., 2018). The use of electron microscopy has determined that memory T cells have altered mitochondrial morphology with fused cristae, that appears to favor OXPHOS and FAO (Buck et al., 2016). Furthermore, a recent study identified a crucial role for CD28 co-stimulatory signals during initial T cell activation to ‘prime’ mitochondria with elevated spare respiratory capacity, that is necessary for the rapid recall responses of memory T cells (Klein Geltink et al., 2017). The ability of quiescent memory T cells to re-acquire effector function rapidly upon TCR triggering is also dependent upon immediate re-engagement of glycolysis (Gubser et al., 2013; Klein Geltink et al., 2017). Therefore, in general terms, a highly glycolytic metabolism is associated with T cell effector responses, whereas low level glycolysis and lipid metabolism is associated with memory and regulatory T cell responses.

## Mechanisms Underpinning the Role of Aerobic Glycolysis in T Cell Function

The function of the Warburg effect in activated effector T cells is incompletely understood. Aerobic glycolysis is an inefficient means of energy production, producing only 2 molecules of ATP as compared to between 30 and 36 produced by OXPHOS. Furthermore, experiments using the ATP synthase inhibitor oligomycin demonstrated that mitochondrial ATP production via OXPHOS is required for initial stages of T cell activation and proliferation (Chang et al., 2013). By contrast, data indicate that expression of the rate-limiting glycolytic enzyme hexokinase 2 (HK2) is actually dispensable for early stages of T cell activation (Tan et al., 2017). It has been suggested that a key advantage of the Warburg effect for cancer cells, and presumably for all proliferating cells, is that it allows the metabolic flexibility required to build biomass (Vander Heiden et al., 2009). Thus, a key function of a switch to aerobic glycolysis might be to enable T cells to use glucose for the generation of biosynthetic precursors for amino acids and nucleic acids, critical for rapid growth, and population expansion, via the pentose phosphate pathway (PPP) (reviewed in (Lunt and Vander Heiden, 2011)). Carbon tracing experiments indicate that, in activated T cells, up to 85% of glucose is excreted as lactate (Fox et al., 2005), indicating that only a minor proportion of glucose-derived carbon is used to fuel biosynthetic pathways. Rather, the NADPH generated by the PPP is rate-limiting in the production of amino acids, nucleic acids, and fatty acids in T cells, and it is likely that aerobic glycolysis allows a faster flux through this pathway as compared to mitochondrial respiration (Vander Heiden et al., 2009). Indeed, blockade of lactate excretion using pharmacological inhibitors of the monocarboxylate transporter MCT1 inhibits T cell proliferation (Murray et al., 2005). Furthermore, a number of recent studies have provided evidence that elevated glucose uptake, and engagement of aerobic glycolysis modulates T cell effector responses through additional mechanisms.

Chang et al. (2013) demonstrated an important role for glyceraldehyde 3-phosphate dehydrogenase (GAPDH) in regulating effector T cell function via a post-transcriptional mechanism. These researchers showed that, in addition to functioning as a glycolytic enzyme, GAPDH binds to the 3′-untranslated region (UTR) of IFNγ mRNA and prevents efficient translation (Chang et al., 2013). By engaging glycolysis, effector T cells sequester GAPDH away from IFNγ mRNA and thereby enhance cytokine production. Further mechanistic insight into the role of glycolysis in inflammatory cytokine production comes from studies of lactate dehydrogenase A (LDHA) function in T cells. LDHA expression is enhanced in activated T cells and is required to support aerobic glycolysis (Peng et al., 2016). Furthermore, IFNγ production was reduced in LDHA-deficient CD4^+^ Th1 cells as compared to control cells, consistent with the known role of glycolysis in T cell effector function. This effect was independent of the *Ifng* 3′-UTR, indicating a distinct effect on cytokine production from that mediated by GAPDH. In the absence of LDHA, histone 3 acetylation at lysine 9 (H3K9Ac) and lysine 27 (H3K27Ac) within the *Ifng* promoter region was substantially decreased (Peng et al., 2016). This glycolysis-dependent epigenetic regulation of IFNγ expression via histone acetylation was mediated by LDHA-dependent maintenance of high levels of acetyl-CoA in effector Th1 cells (Peng et al., 2016).

Studies from the Kaech laboratory revealed a further role for glycolytic flux in T cell activation. Thus, production of the glycolytic metabolite phosphoenolpyruvate (PEP) via enolase promotes prolonged Ca^2+^ responses and activation of the transcription factor nuclear factor of activated T cells (NFAT) (Ho et al., 2015). Nuclear translocation and the transcriptional activity of NFAT regulates the expression of key effector molecules such as IL-2, IFNγ and CD40L in T cells (Hogan, 2017). PEP binds and inhibits the activity of the sarco/endoplasmic reticulum Ca^2+^ ATPase (SERCA), preventing transfer of Ca^2+^ from the cytosol to the SR and prolonging NFAT activation (Ho et al., 2015). Importantly, defects in Ca^2+^/NFAT signaling and T cell activation under conditions of low glucose could be partially corrected by restoration of PEP levels following enforced expression of the gluconeogenesis enzyme PEP carboxykinase 1 (PEPCK1) (Ho et al., 2015). Furthermore, PEPCK1-overexpressing CD4^+^ (Ho et al., 2015) and CD8^+^ (Ma et al., 2018) T cells had elevated anti-tumor responses as compared to control cells, indicating that PEP production serves as a metabolic checkpoint *in vivo*. In NFATc1-deficient T cells, transcript levels of glycolytic proteins such as GLUT1, GLUT3 and HK2 were substantially reduced with a concomitant impairment of glycolytic flux, an effect that could be rescued partially by IL-2 (Klein-Hessling et al., 2017). Therefore NFATc1 regulates T cell activation and upregulation of the glycolytic pathway, which in turn acts in a positive-feedback loop to prolong NFATc1 signaling via PEP.

A novel role for the glycolytic enzyme enolase-1 (Eno1) in inducible Treg function was recently described by Materese and colleagues. These researchers showed that inhibition of glycolysis using 2-DG limited *FOXP3* gene splicing and expression in human Tregs (De Rosa et al., 2015). In Tregs treated with 2-DG, a substantially increased proportion of Eno1 was recruited to the *FOXP3* promoter and regulatory elements, whilst shRNA knockdown of Eno1 expression restored FOXP3 expression (De Rosa et al., 2015). These data suggest that nuclear Eno1 regulates FOXP3 splicing and that engagement of the glycolytic function of Eno1 interferes with this nuclear role, thereby stabilizing the Treg phenotype and function.

A further key role for glucose in T cell activation is to fuel protein O-GlcNacylation. In this pathway, glucose is diverted from the glycolytic pathway (at the level of fructose-6-phosphate) into the hexosamine biosynthetic pathway, which ultimately provides the donor substrate for O-GlcNacylation (Yang and Qian, 2017). TCR triggering results in a substantial increase in the pool of intracellular UDP-GlcNac, resulting in post-translational modification of Ser / Thr residues, and modifying the activity or stability of key proteins, including c-Myc (Swamy et al., 2016). Experiments investigating the impact of T cell-specific deletion of O-GlcNAc transferase (OGT) demonstrated a requirement for this pathway in T cell development in the thymus as well as the clonal expansion of mature T cells (Swamy et al., 2016). Supplementation of *in vitro* T cell cultures with GlcNAc favors Treg differentiation, at the expense of inflammatory Th17 cells, by promoting IL-2R signaling (Araujo et al., 2017). Thus, it is possible that aerobic glycolysis might impinge on Treg differentiation by limiting the supply of metabolites to the hexosamine and O-GlcNAc biosynthetic pathways.

In summary, it is clear that engagement of aerobic glycolysis impacts on T cell function through a number of distinct mechanisms: (i) glycolysis provides a source of ATP and enables the production of biosynthetic precursors to enable proliferation and cell growth; (ii) engagement of the glycolytic pathway and enzymes such as Eno1 and GAPDH diverts their function away from non-glycolytic functions that impinge on T cell gene expression; (iii) glycolytic metabolites such as PEP have additional signaling functions in T cells; (iv) the engagement of glycolysis interacts in a complex network with additional metabolic pathways such as the hexosamine pathway and glutaminolysis to regulate T cell behavior. Further investigation into the function of the Warburg effect in T cells will, no doubt, add to this list of mechanisms in the coming years.

## mTor Regulates T Cell Differentiation

**FIGURE 2:**
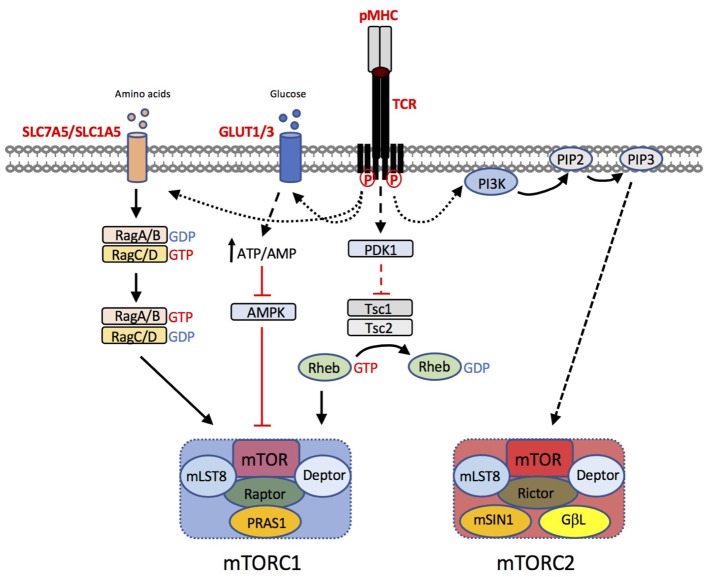
T cell pathways to mTORC1 and mTORC2 activation. T cell receptor (TCR) triggering by peptide (p)MHC complexes results in upregulation of amino acid transporters such as SLC7A5 and SLC1A5. Leucine and glutamine are critical amino acids for the activation of the Rag GTPases, that in turn regulate mTORC1 activation at the lysosome. TCR induced upregulation of glucose transporters GLUT1 and GLUT3 enhances glucose uptake. Increases in the intracellular ATP:AMP ratio as a consequence of increased glucose availability, suppresses the activity of AMP kinase (AMPK), preventing its inhibitory effect on mTORC1 activity. Furthermore, TCR-induced phosphoinositide-dependent kinase (PDK)1, is critical for mTOR activation. PDK1 dependent signals prevent the GTPase activating protein (GAP) activity of the Tsc1/Tsc2 complex. Loss of Tsc GAP activity enables Rheb-GTP to activate mTORC1 through an incompletely understood mechanism. The mechanism of mTORC2 activation is less well understood but is likely to be downstream of PI3K/PIP3-dependent pathways.

The signaling pathways that regulate T cell metabolic reprogramming have been the subject of intense research in the past decade. mTOR is an evolutionarily conserved ser/thr kinase that, in T cells, integrates nutrient sensing and antigen-receptor signaling (reviewed in (Salmond and Zamoyska, 2010, 2011; Powell et al., 2012)). mTOR forms two main signaling complexes, mTORC1 and mTORC2, that differ in their sensitivity to the macrolide inhibitor rapamycin. mTORC1 is composed of mTOR in complex with the adapter protein raptor, mammalian lethal with SEC13 protein 8 (MLST8) and proline-rich Akt substrate (PRAS) 1, an endogenous regulator DEPTOR, and is sensitive to rapamycin. By contrast, mTORC2, composed of mTOR, rictor, GβL, and mammalian stress-activated protein kinase interacting protein 1 (mSIN1), is insensitive to acute inhibition by rapamycin. The pathways that regulate mTOR activation in T cells are summarized in **Figure 2**. In brief, mTORC1 activity is regulated by intracellular amino acids via the nutrient sensing Rag GTPases (Wolfson and Sabatini, 2017). Upon TCR stimulation, T cells upregulate the expression of plasma membrane transporters that enable the uptake of amino acids such as leucine and glutamine from the extracellular environment, that in turn sustain mTORC1 activation. Knockout mouse studies have shown that upregulation of the System L amino acid transporter SLC7A5 (Sinclair et al., 2013) and glutamine-transporter SLC1A5 (Nakaya et al., 2014) are both essential for mTORC1 activity in T cells. Glucose levels also regulate mTORC1 by influencing the activity of the negative regulator AMP kinase (AMPK) (Rolf et al., 2013). Furthermore, recent work has shown that, following TCR signaling, the kinase activity of mTORC1 is activated via the upstream kinase PDK1, in a PI3K/Akt-independent manner (Finlay et al., 2012). In addition, co-stimulation through CD28 and signaling mediated via cytokines such as IL-2 and IL-15 contribute to the magnitude of mTOR activation in T cells (Cornish et al., 2006; Ray et al., 2015). Key downstream targets / effectors of mTORC1 include the translational regulators 4E-binding proteins (4E-BPs) and ribosomal protein S6 kinases (S6Ks). The mechanism by which mTORC2 is activated is less well understood but likely involves PI3K/Akt activity (Zinzalla et al., 2011; Yang et al., 2015). mTORC2 targets include Akt and serum and glucocorticoid-induced protein kinase (SGK).

Whilst the anti-proliferative and immunosuppressive properties of rapamycin have been known for decades, seminal studies published in 2009 determined that mTOR activity also influences T cell effector-memory cell fate decisions *in vivo* (Araki et al., 2009). Thus, rapamycin treatment enhanced the quantity and quality of virus-specific CD8^+^ memory T cells in mice. Knockdown of mTORC1 targets S6K1 and 4E-BPs also impacted upon T cell memory differentiation (Araki et al., 2009). Consistent with these findings, activation of the AMPK1 pathway via metformin or via glucose-deprivation restrains mTOR activity (Pearce et al., 2009; Rolf et al., 2013) and enhances T cell memory. Furthermore, experiments have shown that IL-2 drives high levels of mTOR activation and effector CD8^+^ T cell differentiation, whereas IL-15 drives lower levels of mTOR activation and favors memory cell formation (Cornish et al., 2006; Pipkin et al., 2010; Ray et al., 2015; Richer et al., 2015). Recent studies examining daughter cells from the first cell division following TCR stimulation indicate that mTORC1 activity is asymmetrically inherited (Pollizzi et al., 2016). Importantly, the asymmetric inheritance of mTORC1 influences T cell metabolic capacity and cell fate. Thus, daughter cells with high mTORC1 activity had elevated glycolytic flux and generated T cell populations with enhanced effector capacity, whereas cells with lower mTORC1 activity generated long-lived memory cells (Pollizzi et al., 2016).

Studies using mice with T cell-specific deficiencies in mTOR or with selective ablation or hyperactivation of mTORC1 or mTORC2 signaling pathways have defined an important role for both of these signaling complexes in T cell activation, differentiation and effector function (Delgoffe et al., 2009, 2011; Lee et al., 2010; Yang et al., 2011; Zeng et al., 2013, 2016; Pollizzi et al., 2015). CD4^+^ T cells completely lacking mTOR fail to differentiate into Th1, Th2 or Th17 lineages and instead differentiate preferentially into FOXP3^+^ regulatory T cells (Delgoffe et al., 2009). Nonetheless, whilst mTORC1 signals negatively regulate *de novo* Treg differentiation, the suppressive function of fully differentiated Treg also requires mTORC1 activity (Zeng et al., 2013; Gerriets et al., 2016; Chapman et al., 2018; Sun et al., 2018). T cells deficient in the upstream activator of mTORC1, Rheb, are defective in Th1 and Th17 differentiation (Delgoffe et al., 2011) whilst raptor deficiency also impinges upon Th2 differentiation (Yang et al., 2013). By contrast, deletion of tuberous sclerosis 1 (Tsc1) or Tsc2 results in hyperactive mTORC1 and a subsequent loss of naïve T cell quiescence, indicating that restraining mTOR activity is important for the maintenance of immune homeostasis (Yang et al., 2011; Pollizzi et al., 2015). Rictor deficiency (i.e., loss of mTORC2) has a milder effect on Th1 cell activation *in vivo* as compared to loss of mTORC1 function (Yang et al., 2013) but compromises CD4^+^ Th2 differentiation (Lee et al., 2010). Similarly, Rheb/mTORC1-dependent signals are also required for CD8^+^ T cell differentiation whilst rictor/mTORC2-dependent signals regulate CD8^+^ T cell memory (Pollizzi et al., 2015; Zhang et al., 2016).

Thus, studies of knockout mouse models have shed significant insight into the multifarious roles of mTOR in T cell differentiation and effector function. In this regard, mTORC1 has dual and apparently opposing roles in Treg biology; on the one hand, elevated mTORC1 favors the differentiation of effector T cells at the expense of Tregs, whilst on the other, mTOR expression in Tregs is essential to prevent autoimmunity. Furthermore, these studies have suggested distinct roles for the mTORC1 and mTORC2 complexes in T cells; for example, in Th1 vs. Th2 differentiation. Importantly, evidence indicates that the regulation of T cell metabolism by mTOR complexes is central to these complex phenotypes.

## Regulation of T Cell Metabolism by mTor

In T cells, mTORC1 signaling serves to promote aerobic glycolysis and as a consequence impacts upon T cell differentiation and effector function. Rapamycin treatment substantially impairs the initial TCR-induced upregulation of glucose transporters, glucose uptake and glycolytic enzymes in both CD4^+^ and CD8^+^ T cells (Shi et al., 2011; Finlay, 2012). Similarly, genetic ablation of Rheb (Pollizzi et al., 2015) or raptor (Yang et al., 2013) impairs the upregulation of aerobic glycolysis in TCR-stimulated T cells, whilst hyperactivation of mTORC1 in Tsc1 or Tsc2-deficient T cells is associated with enhanced glycolytic metabolism. Furthermore, mTORC1 activity is required to sustain high levels of aerobic glycolysis in effector T cells (Finlay, 2012; Hukelmann et al., 2016). In this regard, rapamycin treatment caused an approximate 50% reduction in levels of GLUT1 and GLUT3 in IL-2 maintained effector CTLs and a proportional decrease in glucose uptake and lactate production (Hukelmann et al., 2016). By contrast, inhibition of mTORC2 activity actually increases the metabolic capacity of CD8^+^ T cells. Thus, Rictor-deficient CD8^+^ T cells have elevated glycolytic flux, spare respiratory capacity (SRC) and FAO (Pollizzi et al., 2015; Zhang et al., 2016). The mechanism by which deletion of mTORC2 results in increased metabolic fitness has not been fully elucidated but may involve stabilization of nuclear Foxo1 transcription factor (Zhang et al., 2016). Thus, knockdown of Foxo1 reverses the impact of Rictor-deficiency on T cell memory formation whilst expression of a constitutively active Foxo1 in CD8^+^ T cells results in elevated SRC and FAO (Zhang et al., 2016).

The molecular mechanisms by which mTORC1 signals regulate glycolytic pathways in T cells are also incompletely understood. Studies have identified transcription factors including Myc (Wang et al., 2011) and hypoxia inducible factor 1 alpha (HIF-1α) (Shi et al., 2011; Finlay et al., 2012) as key drivers of metabolic reprogramming in T cells. Myc-deficient T cells are defective in TCR-induced upregulation of glucose transporters and glycolytic enzymes and have substantially reduced glycolytic flux (Wang et al., 2011). HIF-1α is upregulated strongly in Th17 cells (Shi et al., 2011) and effector CD8^+^ T cells (Finlay et al., 2012) and, similar to Myc, is important for the upregulation of aerobic glycolysis in these T cell subsets. Importantly, rapamycin impairs the TCR-induced expression of both Myc and HIF-1α (Shi et al., 2011; Wang et al., 2011; Finlay et al., 2012; Pollizzi et al., 2016) indicating that mTOR serves to regulate aerobic glycolysis, at least in part, through regulation of Myc and HIF-1α expression and their subsequent downstream transcriptional programs. mTORC1 has been reported to regulate Myc expression via post-transcriptional mechanisms as levels of Myc protein, but not mRNA, were reduced in Raptor-deficient T cells as compared to controls (Yang et al., 2013).

In addition to the role of mTOR in promoting glycolytic metabolism in effector T cells, recent evidence has shown that mTOR also has a vital role in the regulation of mitochondrial metabolism. Gene expression and pathway analysis identified the regulation of both glycolysis and OXPHOS as being significantly impacted by hyperactive mTOR activity in *Tsc1^−/−^* T cells (Shrestha et al., 2014). Furthermore, mTOR catalytic site inhibitors reduced expression of mitochondrial and OXPHOS genes in activated Tregs (Chapman et al., 2018). In both cases, mTOR activity was required for the transcriptional programs driving OXPHOS in T cells, highlighting the dual role for this kinase in regulating mitochondrial and glycolytic metabolism.

## Activated mTor Leads to Altered T Cell Metabolism in Autoimmunity

The use of rapamycin/sirolimus as an immunosuppressive agent in the clinic was approved by the US FDA in 1999. Rapamycin and its derivatives have been used extensively in transplantation to limit organ rejection, however, recent evidence has shown that these compounds may have broader applicability in the treatment of cancers and autoimmunity, as well as in vaccine design (reviewed in Perl, 2015, 2016). Importantly, the clinical benefits of mTOR blockade in inflammatory diseases has been linked to the modulation of T cell metabolism. For example, inflammatory T cells from systemic lupus erythematosus (SLE) patients have substantially elevated glycolytic and mitochondrial metabolism (Yin et al., 2015) and mTOR activity (Kato and Perl, 2014), as compared to healthy controls. Importantly, in both mouse models and in human patients, T cell metabolism and inflammatory cytokine production could be normalized by reducing mTOR activity through metformin or rapamycin treatment (Kato and Perl, 2014; Yin et al., 2015). Furthermore, and consistent with an important role for mTOR-driven inflammatory T cells in the pathogenesis of lupus, a recent phase 1/2 trial reported that rapamycin had a beneficial impact on clinical disease scores in a cohort of 43 SLE patients (Lai et al., 2018). Improved disease outcomes were associated with decreased inflammatory T cell activity and increased Treg numbers (Lai et al., 2018), consistent with the known role of mTOR in regulating T cell metabolism and effector responses.

## Concluding Remarks

Our understanding of the close links between the regulation of aerobic glycolysis and T cell function has been transformed in the past decade. Furthermore, mTOR signals have emerged as a key driver of these processes. As our understanding of the molecular details and signaling pathways leading to metabolic reprogramming increases, then the opportunity to translate these findings into the clinic should emerge. In this regard, evidence for distinct roles for mTORC1 and mTORC2 in modulating T cell metabolism and activation gives scope for more precise manipulation of these pathways in the future.

## Author Contributions

RS wrote the manuscript and generated the figures.

## Conflict of Interest Statement

The author declares that the research was conducted in the absence of any commercial or financial relationships that could be construed as a potential conflict of interest.
